# Mechanical behavior of primary cilia

**DOI:** 10.1186/2046-2530-1-S1-P27

**Published:** 2012-11-16

**Authors:** CR Jacobs, ME Downs, AM Nguyen, FA Herzog, DA Hoey

**Affiliations:** 1 Columbia University, NY, USA; 2 Ecole Polytechnique Federale de Lausanne, Switzerland; 3 Trinity Collage Dublin, Ireland

## 

We have developed a novel combined experimental/modeling approach to determine the mechanical properties of primary cilia. Specifically, we developed a large rotation formulation of cilia bending with fluid flow that accounts for rotation at the base of the cilium, the initial shape of the cilium and fluid drag at high deflection angles. We also have obtained three dimensional configurations of primary cilia in their deformed configuration at 3Hz acquired with high-speed confocal microscopy. We found a wide variety of previously unreported bending shapes and behaviors. Cilia appear to deflect in smooth bending shapes, rigid-body rotation, and in a hinged or kinked pattern. This suggests that both the axoneme and basal body anchorage are important to understanding deflection patterns. We also analyzed post-flow relaxation patterns. These results indicate that with removal of force, cilia slowly return to their initial configuration. However, they also can return to a different configuration with “overcorrection” observed that suggests adaptation of mechanical properties. Results from our combined experimental and theoretical approach suggest that the average flexural rigidity of primary cilia might be higher than previously reported. In addition our findings indicate the mechanics of primary cilia are potentially non-linear, richly varied, and mechanisms may exist to alter their mechanical behavior.

